# Kinetic study of lipase-catalyzed glycerolysis of palm olein using Lipozyme TLIM in solvent-free system

**DOI:** 10.1371/journal.pone.0192375

**Published:** 2018-02-05

**Authors:** Thomas Shean Yaw Choong, Chiou Moi Yeoh, Eng-Tong Phuah, Wai-Lin Siew, Yee-Ying Lee, Teck-Kim Tang, Luqman Chuah Abdullah

**Affiliations:** 1 Department of Chemical and Environmental Engineering, Faculty of Engineering, Universiti Putra Malaysia, Serdang, Selangor, Malaysia; 2 INTROP, Universiti Putra Malaysia, Serdang, Selangor, Malaysia; 3 Department of Agriculture and Food Science, Faculty of Science, Universiti Tunku Abdul Rahman, Kampar, Perak, Malaysia; 4 Malaysian Palm Oil Board, Persiaran Institusi, Bandar Baru Bangi, Kajang, Selangor, Malaysia; 5 Institute of Bioscience, Universiti Putra Malaysia, Serdang, Selangor, Malaysia; 6 School of Science, Monash University Malaysia, Bandar Sunway, Selangor, Malaysia; MJP Rohilkhand University, INDIA

## Abstract

Diacylglycerol (DAG) and monoacylglycerol (MAG) are two natural occurring minor components found in most edible fats and oils. These compounds have gained increasing market demand owing to their unique physicochemical properties. Enzymatic glycerolysis in solvent-free system might be a promising approach in producing DAG and MAG-enriched oil. Understanding on glycerolysis mechanism is therefore of great importance for process simulation and optimization. In this study, a commercial immobilized lipase (Lipozyme TL IM) was used to catalyze the glycerolysis reaction. The kinetics of enzymatic glycerolysis reaction between triacylglycerol (TAG) and glycerol (G) were modeled using rate equation with unsteady-state assumption. Ternary complex, ping-pong bi-bi and complex ping-pong bi-bi models were proposed and compared in this study. The reaction rate constants were determined using non-linear regression and sum of square errors (SSE) were minimized. Present work revealed satisfactory agreement between experimental data and the result generated by complex ping-pong bi-bi model as compared to other models. The proposed kinetic model would facilitate understanding on enzymatic glycerolysis for DAG and MAG production and design optimization of a pilot-scale reactor.

## Introduction

The prevalence of overweight and obesity owing to the imbalance between energy intake and energy expenditure has triggered attention to the exploitation of an effective approach such as the development of structured-lipid, starch or protein-based fat replacer as well as food supplements and medication, to reverse the fasting growing obesity trend. Recent disclosure of diacylglycerol (DAG)-enriched oil has therefore become a subject of growing interest worldwide due to its anti-obesity properties. Previous research findings revealed that DAG possesses the ability to reduce serum triacylglycerol (TAG), enhance β-oxidation activity and prevent abnormal accumulation of fat [1–3]. In addition, DAG in synergistic with monoacylglycerol (MAG) can act as excellent emulsifiers and stabilizers in food and pharmaceutical industry [4]. MAG alone has also been widely employed in textile processing, production of plastics and formulation of oil for machinery on account of its excellent lubricant and plasticizing properties. Therefore, the demand for both DAG and MAG has skyrocketed in recent years [5–7].

Mixture of DAG and MAG can be produced chemically. However, the major barrier of the chemical process is extreme operating condition required to accelerate the overall reaction rate with the use of an inorganic catalyst. This eventually results in the formation of undesired by-products (oxidized compounds and soap) and purification of products is therefore needed [8]. The breakthrough in enzyme technology in fats and oils industry for the modification of edible oil has drawn attentions worldwide. Enzymatic approach provides an alternative option due to its mild processing conditions, high regioselectivity of lipases and less environmental impact [9, 10]. Various enzyme-mediated methods such as hydrolysis of TAG [11–13], glycerolysis of TAG [14–17], esterification of fatty acids and glycerol [11, 15] have been reported for DAG and MAG manufacturing. Among these enzymatic methods, glycerolysis of TAG had been proven to be efficient for the preparation of DAG and MAG due to the huge amounts of glycerol surplus from biodiesel industries as cheap reactants and high production yield [18–20].

Earlier we have analyzed enzymatic glycerolysis for DAG production, employing different commercial enzymes namely Lipozyme RMIM, Lipozyme TLIM and Novozyme 435. Lipozyme TLIM was found to surpass others on cost performance basis as it is cheaper and it showed greater affinity towards DAG synthesis which translates to high feasibility of commercialization [17]. Kinetic evaluation and simulation of biochemical process is important in the design of scale-up experiment in order to gain an insight into reaction mechanism of enzymatic glycerolysis. Ping-pong bi-bi and ordered-sequential bi-bi mechanisms are the common mathematical models in the literatures used to represent the lipase-catalyzed reaction regardless of the type of bioconversion [13, 20–28]. Nevertheless, to the best of our knowledge, kinetic evaluation of lipase-catalyzed glycerolysis of palm olein in solvent-free system remains vague Therefore, in this paper, three kinetic models namely simple ternary complex, simple ping-pong bi-bi and complex ping-pong bi-bi were proposed based on unsteady-state assumption to simulate the overall glycerolysis process and comparison among the kinetic mechanisms was emphasized.

## Materials and methods

### Materials

Palm olein IV 56 was purchased from Sime Darby, Malaysia; glycerol anhydrous (99.9%) was purchased from Mallinckrodt Baker, USA; and Lipozyme TL IM was given by Novozymes A/S, Denmark. Acetone from Fisher Scientific UK Limited, UK and acetonitrile from Mallinckrodt Baker, USA were high performance liquid chromatography (HPLC) grade. All other chemicals used were reagent grade. Standards for HPLC (1,2-dipalmitin, 1,3-dipalmitin, 1,2-diolein, 1,3-distearin, 1,2-dimyristin, 1,3-dilinolein, 2-oleoylglyerol, 1,2-dipalmitoyl-3-oleoyl-rac-glycerol) were purchased from Sigma Chemical, USA, while other standards (1,2-diolein, 1,3-diolein, glycerol-1-palmitate-3-oleate, glycerol-1-palmitate-2-oleate) were purchased from Larodan, Sweden. Monopalmitin and monoolein were purchased from Tokyo Kasei, Japan.

### Glycerolysis reaction

Enzymatic glycerolysis reaction was conducted in solvent-free system as described in earlier work [17]. The reactions were performed by reacting 100 g palm olein in the presence of enzyme at glycerol to enzyme (G/E) mass ratio of 1 in a 250 ml conical flask. Three enzyme loads (3, 5 and 8 wt % oil) were studied. The reaction mixture was incubated at 55°C and shaken at 200 rpm in a water bath shaker. Samples were taken at specific time intervals where each sample was stored at -20°C prior to analysis. All reactions were carried out in duplicate.

### Reversed-Phase HPLC analysis

Acylglycerol compositions were determined by high-performance liquid chromatography (HPLC) (Gilson, France) equipped with a refractive index detector model 2410 (Waters, USA), using two commercially packed LiChrospher®100 RP-18 column end capped (250mm) with 5 μm particle size in series. The glyceride compositions were eluted with acetone/acetonitrile (70:30) for TAG determination and acetone/acetonitrile (50:50) for free fatty acid (FFA), MAG and DAG determination, both at a flow rate of 1 ml/min. The TAG and DAG were identified according to Swe [29] and Ghazali [30]. Calibration curves were constructed, and the results were given as the weight ratio of total glycerides.

### Kinetic modeling

In present work, a time course analysis was conducted to determine the rate constants for each reaction. The rate constants of forward and backward reaction were determined individually so that the effect of each step on lipase-catalyzed glycerolysis process could be better understood.

Three models were considered in this study:

- Ternary complex model;
- Simple ping-pong bi-bi model; and
- Complex ping-pong bi-bi model

A material balance was performed for each component involved in the model. Two assumptions were made while performing the material balance:

- Mass transfer limitation in the reaction system was negligible (both internal and external mass transfer resistance can be ignored when considering Lipozyme IM with porous support and sufficient stirring or shaking speed and;
- All the reactions were elementary.
- No loss of enzyme activity throughout the reaction
- No significant amount of free fatty acid is formed as the glycerolysis was carried out in micro-aqueous environment.

#### (A) Simple ternary complex model

Referring to Eqs 1–3, simple ternary model proposed in this study suggested that enzymatic glycerolysis reaction follows ordered-sequential bi-bi mechanism in which substrate TAG was initially bound to the enzyme (E) to form a binary complex (E.TAG). Glycerol (G) was then attached to the binary complex before any reaction takes place to form intermediate product of ternary complex (E.G.TAG). Instantaneously, products DAG and MAG were formed and released with restoration of enzyme particles.

$$\mathrm{TAG}+E\;\overset{k_{1}}{\underset{k_{2}}{⇌}}\;E.\mathrm{TAG}$$ (1)

$$E.\mathrm{TAG}+G\;\overset{k_{3}}{\underset{k_{4}}{⇌}}\;E.G.\mathrm{TAG}$$ (2)

$$E.G.\mathrm{TAG}\;\overset{k_{5}}{\to }\;\mathrm{DAG}+\mathrm{MAG}+E$$ (3)

where k_1_ is the forward reaction rate for TAG and E to form binary complex (E.TAG); k_2_ is the reverse reaction of E.TAG complex to cleave into TAG and E; k_3_ indicates the forward reaction rate for E.TAG complex to form ternary complex (E.G.TAG); k_4_ represents the reverse process rate for ternary complex to reform binary complex; k_5_ is forward reaction for DAG and MAG formation.

Considering the steps described in Eqs 1–3 as elementary reactions, the rate equation for the substrates, products and complexes were given as:

$$\frac{d[TAG]}{dt}=k_{2}\;[E.TAG]-k_{1}\;[E][TAG]$$ (4)

$$\frac{d[DAG]}{dt}=k_{5}\;[E.G.TAG]$$ (5)

$$\frac{d[MAG]}{dt}=k_{5}\;[E.G.TAG]$$ (6)

$$\frac{d[G]}{dt}=k_{4}\;[E.G.TAG]-k_{3}\;[E.TAG][G]$$ (7)

$$\frac{d[E]}{dt}=k_{2}\;[E.TAG]+k_{5}\;[E.G.TAG]-k_{1}\;[E][TAG]$$ (8)

$$\frac{d[E.TAG]}{dt}=k_{1}\;[E][TAG]-k_{4}\;[(E.TAG).G]-k_{2}\;[E.TAG]-k_{3}\;[E.TAG][G]$$ (9)

$$\frac{d[E.G.TAG]}{dt}=k_{3}\;[E.TAG][G]-k_{4}\;[E.G.TAG]$$ (10)

The sum of all the intermediate enzyme complexes is equal to the enzyme loading as shown in the following equation:

$$E_{0}=[E]+[E.\mathrm{TAG}]+[E.G.\mathrm{TAG}]$$ (11)

#### (B) Simple ping-pong bi-bi model

Simple ping-pong bi-bi model was also adopted to simulate the kinetic mechanism of enzymatic glycerolysis (Eq 12–15). Binding of the substrate TAG to enzyme (E) to form a binary complex (E.TAG) is the initial step of the reaction, followed by the formation of DAG and a modified enzyme complex (E.FFA). Glycerol (G) is then bound to the modified enzyme complex (E.FFA) forming another binary complex (E.FFA).G. Finally, MAG is released and the enzyme is regenerated.

$$\mathrm{TAG}+E\;\overset{k_{1}}{\underset{k_{2}}{⇌}}\;E.\mathrm{TAG}$$ (12)

$$E.\mathrm{TAG}\;\overset{k_{3}}{\to }\;\mathrm{DAG}+E.\mathrm{FFA}$$ (13)

$$E.\mathrm{FFA}+G\;\overset{k_{4}}{\underset{k_{5}}{⇌}}\;(E.\mathrm{FFA}).G$$ (14)

$$(E.\mathrm{FFA}).G\;\overset{k_{6}}{\to }\;\mathrm{MAG}+E$$ (15)

where k_1_ and k_2_ relate to forward reaction coefficient for binary complex (E.TAG) formation and reverse rate constant for TAG and E reformation, respectively; k_3_ is the forward reaction of E.TAG to form main product DAG coinciding with the formation of modified enzyme complex (E.FFA); k_4_ indicates the reaction between E.FFA complex and G to form secondary binary complex (E.FFA).G; k_5_ shows the reverse process which will form E.FFA and G; k_6_ is forward reaction coefficient for MAG formation. The entire rate equation for the substrates, products, and complexes were represented as:

$$\frac{d[TAG]}{dt}=k_{2}\;[E.TAG]-k_{1}\;[E][TAG]$$ (16)

$$\frac{d[DAG]}{dt}=k_{3}\;[E.TAG]$$ (17)

$$\frac{d[MAG]}{dt}=k_{6}\;[(E.FFA].G]$$ (18)

$$\frac{d[G]}{dt}=k_{5}\;[(E.FFA).G]-k_{4}\;[E.FFA][G]$$ (19)

$$\frac{d[E]}{dt}=k_{2}\;[E.TAG]+k_{6}\;[(E.FFA).G]-k_{1}\;[E][TAG]$$ (20)

$$\frac{d[E.TAG]}{dt}=k_{1}\;[E][TAG]-k_{2}\;[E.TAG]-k_{3}\;[E.TAG]$$ (21)

$$\frac{d[E.FFA]}{dt}=k_{3}\;[E.TAG]+k_{5}\;[(E.FFA).G]-k_{4}\;[E.FFA][G]$$ (22)

$$\frac{d[(E.FFA).G]}{dt}=k_{4}\;[E.FFA][G]-k_{5}\;[(E.FFA).G]-k_{6}\;[(E.FFA).G]$$ (23)

The sum of all the intermediate enzyme complexes is equal to the enzyme loading as shown in the following equation:

$$E_{0}=[E]+[E.\mathrm{TAG}]+[E.\mathrm{FFA}]+[(E.FFA).G]$$ (24)

#### (C) Complex ping-pong bi-bi model

The complex ping-pong bi-bi model is an extension of the simple ping-pong bi-bi model where this mechanism suggests that some of the diacylglycerol produced become a substrate to the enzyme (E) forming a binary complex (E.DAG), followed by the release of monoacylglycerol (MAG) and a modified enzyme complex (E.FFA) as shown.

$$\mathrm{TAG}+E\;\overset{k_{1}}{\underset{k_{2}}{⇌}}\;E.\mathrm{TAG}$$ (25)

$$E.\mathrm{TAG}\;\overset{k_{3}}{\to }\;\mathrm{DAG}+E.\mathrm{FFA}$$ (26)

$$E.\mathrm{FFA}+G\;\overset{k_{4}}{\underset{k_{5}}{⇌}}\;(E.\mathrm{FFA}).G$$ (27)

$$(E.\mathrm{FFA}).G\;\overset{k_{6}}{\to }\;\mathrm{MAG}+E$$ (28)

$$\mathrm{DAG}+E\;\overset{k_{7}}{\underset{k_{8}}{⇌}}\;E.\mathrm{DAG}$$ (29)

$$E.\mathrm{DAG}\;\overset{k_{9}}{\to }\;\mathrm{MAG}+E.\mathrm{FFA}$$ (30)

where k_1_ to k_6_ are constants for the reactions as described in simple ping-pong bi-bi model; k_7_ and k_8_ are both the forward and reverse reactions of enzyme complex E.DAG; k_9_ indicates the formation of MAG and E.FFA complex which will react with G for the production of MAG. The rate equation for the substrates, products, and complexes were given as:

$$\frac{d[TAG]}{dt}=k_{2}\;[E.TAG]-k_{1}\;[E][TAG]$$ (31)

$$\frac{d[DAG]}{dt}=k_{3}\;[E.TAG]+k_{8}\;[E.DAG]-k_{7}\;[E][DAG]$$ (32)

$$\frac{d[MAG]}{dt}=k_{6}\;[(E.FFA).G]-k_{9}\;[E.DAG]$$ (33)

$$\frac{d[G]}{dt}=k_{5}\;[(E.FFA).G]-k_{4}\;[E.FFA][G]$$ (34)

$$\frac{d[E]}{dt}=k_{2}\;[E.TAG]+k_{6}\;[(E.FFA].G]+k_{8}\;[E.DAG]-k_{1}\;[E][TAG]-k_{7}\;[E][DAG]$$ (35)

$$\frac{d[E.TAG]}{dt}=k_{1}\;[E][TAG]-k_{2}\;[E.TAG]-k_{3}\;[E.TAG]$$ (36)

$$\frac{d[E.FFA]}{dt}=k_{3}\;[E.TAG]+k_{5}\;[(E.FFA).G]+k_{9}\;[E.DAG]-k_{4}\;[E.FFA][G]$$ (37)

$$\frac{d[E.DAG]}{dt}=k_{7}\;[E][DAG]-k_{8}\;[E.DAG]-k_{9}\;[E.DAG]$$ (38)

$$\frac{d[(E.FFA).G]}{dt}=k_{4}\;[E.FFA][G]-k_{5}\;[(E.FFA).G]-k_{6}\;[(E.FFA).G]$$ (39)

The sum of all the intermediate enzyme complexes is equal to the enzyme loading as shown in the following equation:

$$E_{0}=[E]+[E.\mathrm{TAG}]+[E.\mathrm{FFA}]+[(E.FFA].G]+[E.DAG]$$ (40)

### Parameter estimation

The rate constant parameters were determined by fitting the model equations into experimental data using software Matlab 7.1. Non-linear regression was used to determine the rate constant parameters by minimizing the sum of square errors (SSE) (Eq 41). Root-mean-square deviation (RMSD) and Chi-squared test are also crucial tools used to evaluate the goodness-of-fit between both experimental results and model-generated data (Eqs 42 and 43). High-order Runge-Kutta 4,5 was used to solve the ordinary differential equation (ODE). The rate constants obtained from the non-linear regression may be sensitive to initial guesses. At least three different initial guesses were used to ensure the consistency of the rate constants.

$$SSE=\frac{1}{N}\sum_{j=1}^{N}(X_{exp,j,i}-X_{calc,j,i}{)}^{2}$$ (41)

$$RMSD=[\frac{1}{N}\sum_{j=1}^{N}(X_{calc,j,i}-X_{exp,j,i}{)}^{2}{]}^{1/2}$$ (42)

$$\chi^{2}=\frac{\sum_{j=1}^{N}(X_{exp,j,i}-X_{calc,j,i}{)}^{2}}{N-Z}$$ (43)

where *X*_*calc*,*j*,*i*_ is the calculated weight fraction of species *i* at *j* number of data; *X*_*exp*,*j*,*i*_ is the experimental weight fraction of species *i* at *j* number of data, and *N* and Z are the number of experimental data and number of constants used in the proposed kinetic models, respectively.

## Results and discussion

The rate constants were initially estimated using experimental data of 3 wt-% enzyme load with assumption being made on the independence of the reaction coefficient on enzyme load. The values of the rate constants and statistical evaluation of kinetic models with respect to experimental data are tabulated in Tables 1 and 2, respectively. Statistical analysis in kinetic modeling is a powerful tool for model discrimination as it enables elucidation of the reliability and accuracy of the estimating equations for distinct kinetic models. As depicted in Table 2, SSE, RMSD and chi-square χ^2^ for both 3 wt % and 5 wt % enzyme loads as tested with the three models proposed were found to approach zero, indicating tight fit of the models proposed to the data. Nevertheless, the three models showed relatively high SSE and RMSD as enzyme concentration increased to 8 wt % because excessive enzyme may result in enzyme aggregation, rendering the exposure of the enzyme active site to substrate molecules [20] and therefore improvement and re-estimation of parameters is required.

**Table 1:** Estimated rate constants for the models.

Rate constant (hour –^1^ g^– 1^ enzyme)	Simple Ternary Model	Simple Ping-Pong Bi-Bi Model	Complex Ping-Pong Bi-Bi Model
k_1_	0.011	0.011	0.011
k_2_	0.025	0.025	0.025
k_3_	10.35	0.35	2.5
k_4_	18.81	18.81	1.1
k_5_	6.02	0.01	0.01
k_6_	-	10.92	1.2
k_7_			0.05
k_8_			2.8
k_9_			1.2

(Based on 3 wt-% enzyme load)

**Table 2:** Statistical evaluation of models proposed for different enzyme loads.

Model	Enzyme Load	Sum of Squares Errors (SSE)				Root-mean-square Deviation (RMSD)				Chi-Squared (χ^2^)			
	(wt-%)	TAG	DAG	MAG	Total	TAG	DAG	MAG	Total	TAG	DAG	MAG	TOTAL
Simple Ternary Complex Model	3	0.0028	0.0107	0.0025	0.0160	0.0529	0.1034	0.0500	0.2063	4.6 x 10^−4^	3.1 x 10^−3^	6.9 x 10^−4^	4.25 x 10^−3^
	5	0.0015	0.0079	0.0099	0.0193	0.0387	0.0889	0.0995	0.2271	1.3 x 10^−4^	2.4 x 10^−3^	1.7 x 10^−3^	4.23 x 10^−3^
	8	0.0589	0.0918	0.1990	0.3498	0.2427	0.3030	0.4461	0.9918	8.7 x 10^−3^	1.7 x 10^−2^	4.1 x 10^−2^	6.67 x 10^−2^
Simple Ping-Pong Bi-Bi Model	3	0.0019	0.0014	0.0074	0.0107	0.0436	0.0374	0.0860	0.1670	6.3 x 10^−4^	4.8 x 10^−4^	2.4 x 10^−3^	3.51 x 10^−3^
	5	0.0004	0.0029	0.0164	0.0197	0.0200	0.0539	0.1281	0.2020	1.2 x 10^−4^	9.5 x 10^−4^	5.4 x 10^−3^	6.47 x 10^−3^
	8	0.0362	0.0153	0.0092	0.0607	0.1903	0.1237	0.0959	0.4099	1.2 x 10^−2^	5.0 x 10^−3^	3.0 x 10^−3^	1.28 x 10^−2^
Complex Ping-Pong Bi-Bi Model	3	0.0021	0.0020	0.0024	0.0066	0.0458	0.0447	0.0490	0.1395	1.1 x 10^−3^	1.0 x 10^−3^	1.2 x 10^−3^	3.30 x 10^−3^
	5	0.0004	0.0004	0.0020	0.0028	0.0200	0.0200	0.0447	0.0847	1.8 x 10^−4^	1.9 x 10^−4^	1.3 x 10^−3^	1.67 x 10^−3^
	8	0.0418	0.0067	0.0290	0.0775	0.20445	0.0819	0.1703	0.4566	2.0 x 10^−2^	3.3 x 10^−3^	5.4 x 10^−3^	2.87 x 10^−2^

**Fig 1:**
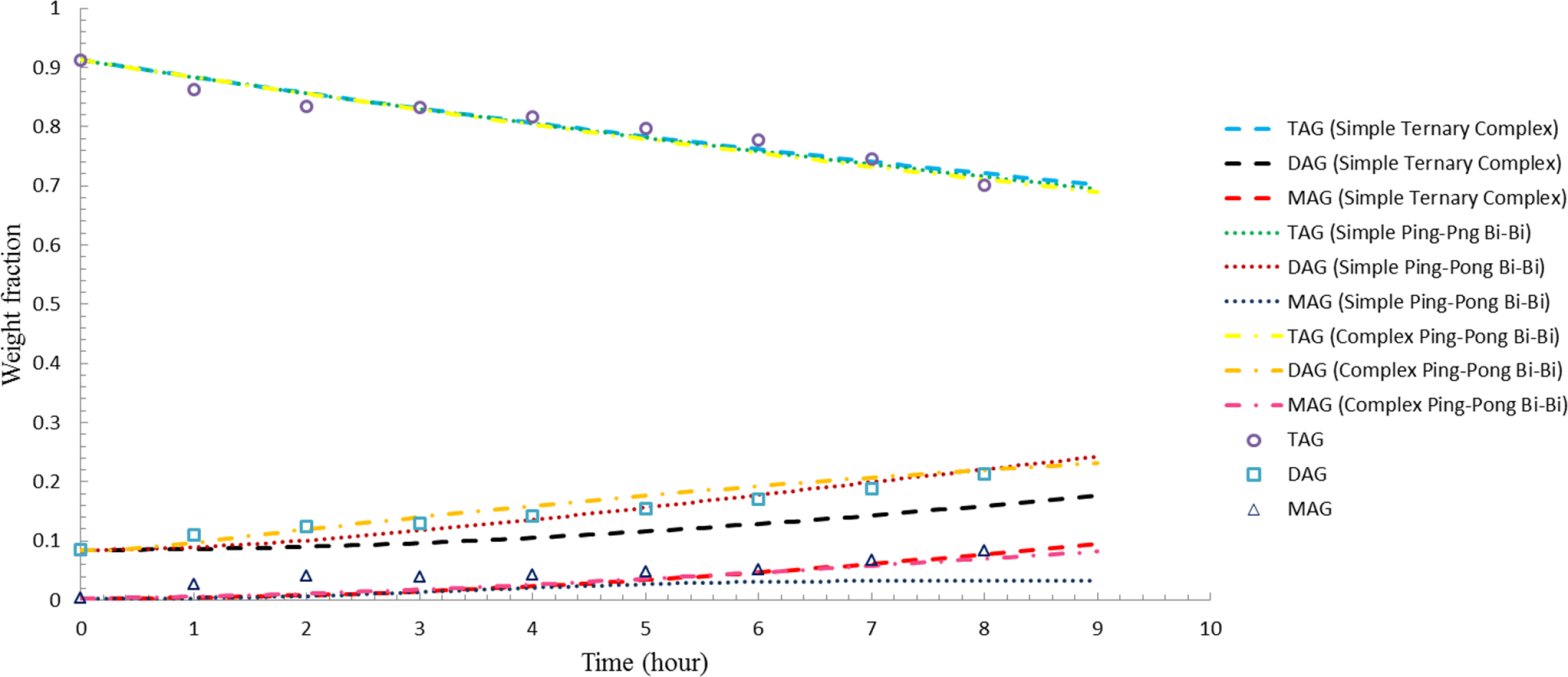
Comparison between simulated results of three different models and experimental data for 3 wt-% enzyme load.

**Fig 2:**
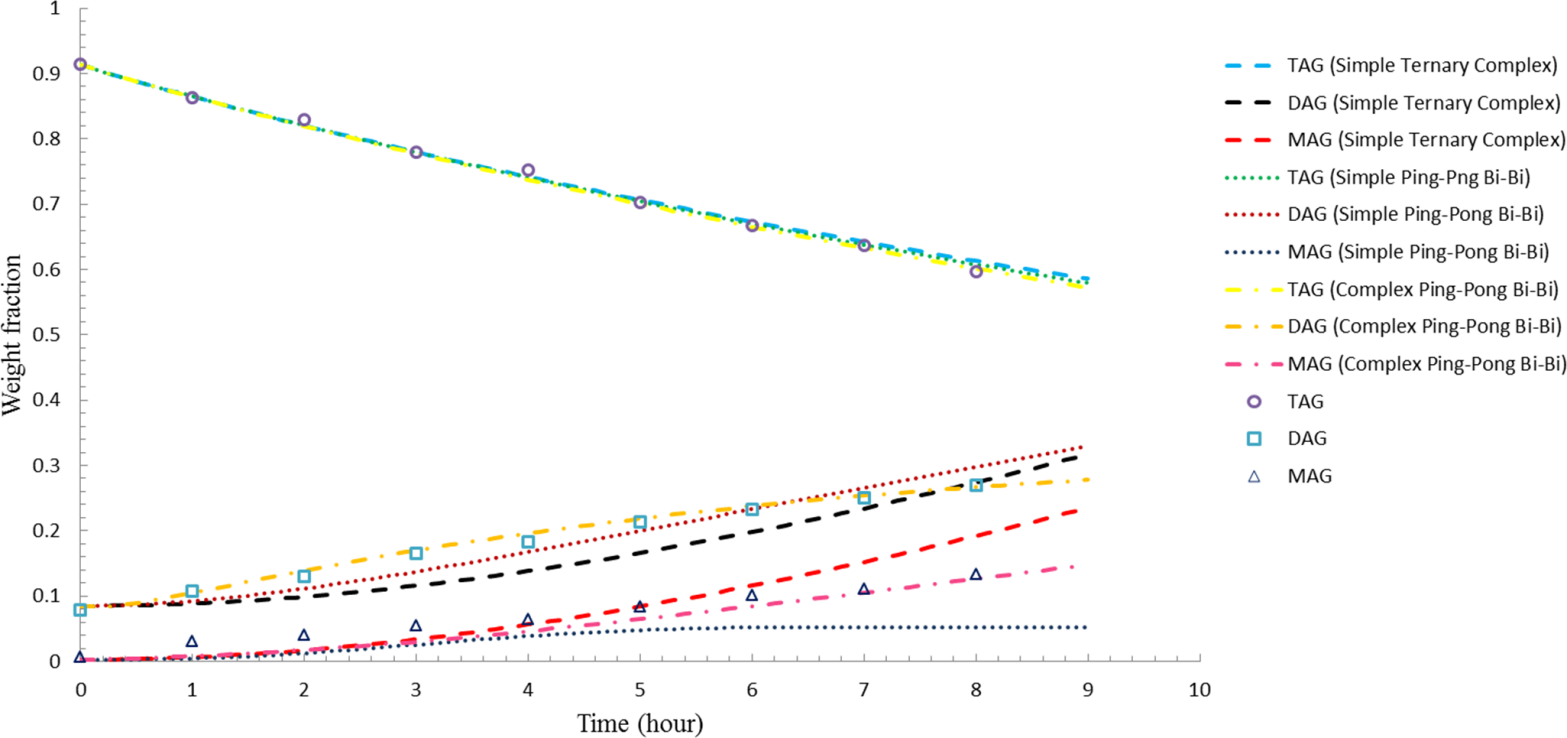
Comparison between simulated results of three different models and experimental data for 5 wt-% enzyme load.

**Fig 3:**
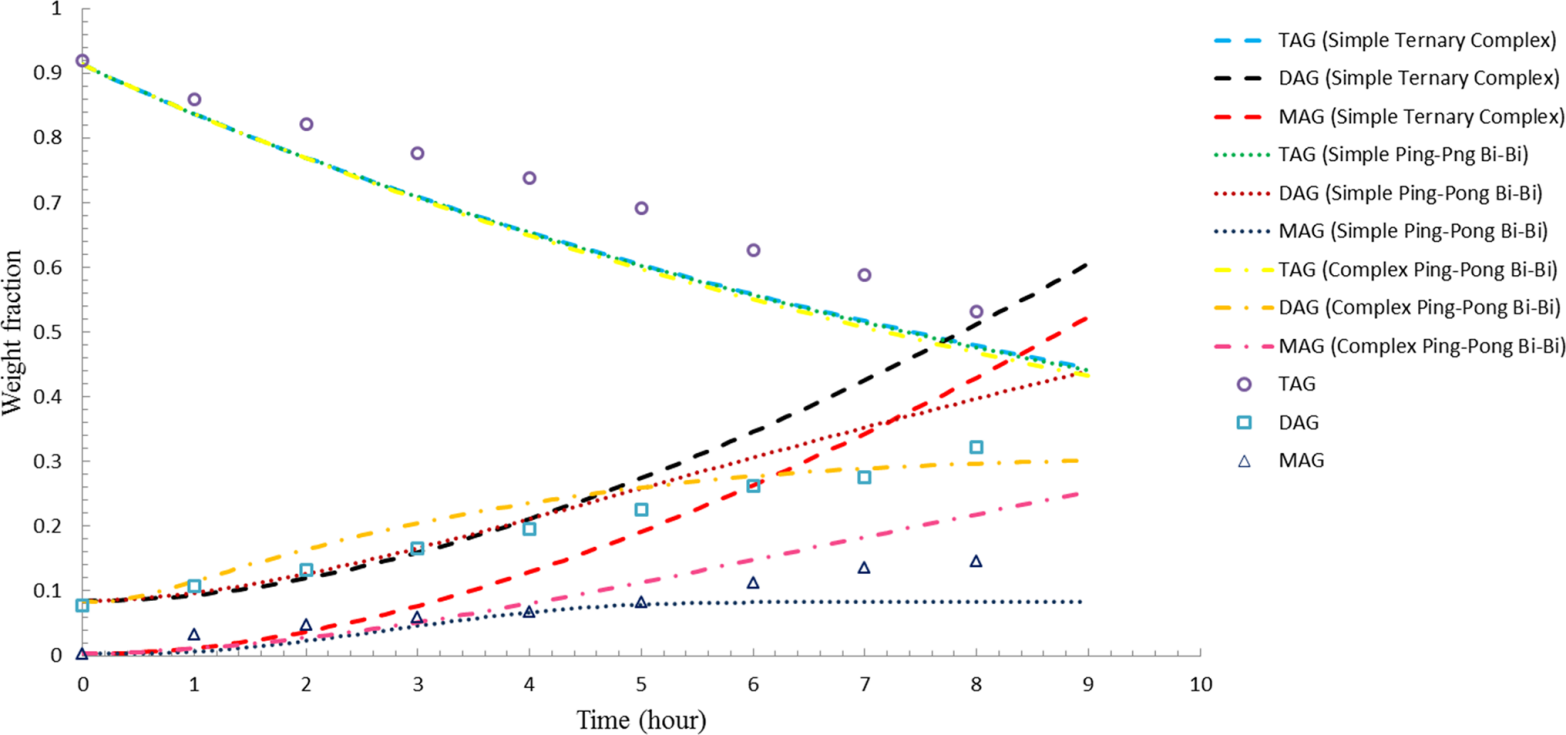
Comparison between simulated results of three different models and experimental data for 8 wt-% enzyme load.

Figs 1–3 illustrate the comparisons between the simulated results and experimental data for enzyme load of 3 wt %, 5 wt %, and 8 wt %, respectively. Even though all models accurately predict the TAG composition for both 3 wt % and 5 wt % enzyme load, complex ping-pong bi-bi model was found to be able to provide a better estimation on the experimental data with regard to DAG and MAG for reactions treated with 3 wt % and 5 wt % enzyme load as opposed to simple ping-pong bi-bi and ternary complex model (Figs 1 and 2). Based on the statistical data, complex ping-pong bi-bi model was considered as the preferable model as it gave the lowest SSE and RMSD for the two enzyme loads tested (Table 2). However, all models (based on the rate constants obtained using 3 wt-% enzyme load) obviously predict fairly well the acylglycerol compositions at 8 wt % enzyme load, as illustrated in Fig 3. The results agreed with Al-Zuhair’s [24] hypothesis that low enzyme concentration model was not suitable for simulating process with high enzyme concentration as the model tends to diverge at high enzyme load.

A new set of rate constants were then revised for the 8 wt-% enzyme load as shown in Table 3. Since the active area of the enzyme was less utilized for TAG at high enzyme concentration as compared to low enzyme concentration, the binding rate between TAG and E, k_1_, was significantly reduced from 0.012 to 0.008 hour –^1^ g^– 1^ enzyme.

**Table 3:** Re-estimated rate constants for the models for 8 wt-% enzyme load.

		Simple Ternary Complex Model	Simple Ping-Pong Bi-Bi Model	Complex Ping-Pong Bi-Bi Model
**Rate constant** **(hour –**^**1**^ **g**^**– 1**^ **enzyme)**	k_1_	0.008	0.008	0.008
	k_2_	0.025	0.025	0.025
	k_3_	10.35	0.35	3.5
	k_4_	18.81	18.81	1.1
	k_5_	5.02	0.01	0.01
	k_6_		10.92	1.2
	k_7_			0.05
	k_8_			4.8
	k_9_			1.2
SSE	TAG	0.0032	0.0028	0.0026
	DAG	0.0064	0.0015	0.0027
	MAG	0.0343	0.0102	0.0037
	Total	0.0439	0.0145	0.0090
RMSD	TAG	0.0567	0.0529	0.0510
	DAG	0.0800	0.0387	0.0520
	MAG	0.1852	0.1001	0.0608
	Total	0.3219	0.1917	0.1638
Chi-squared (χ^2^)	TAG	7.9 x 10^−4^	9.4 x 10^−4^	1.3 x 10^−3^
	DAG	1.6 x 10^−3^	5.1 x 10^−4^	1.4 x 10^−3^
	MAG	8.6 x 10^−3^	3.4 x 10^−3^	1.1 x 10^−3^
	Total	1.1 x 10^−2^	4.85 x 10^−3^	3.8 x 10^−3^

**Fig 4:**
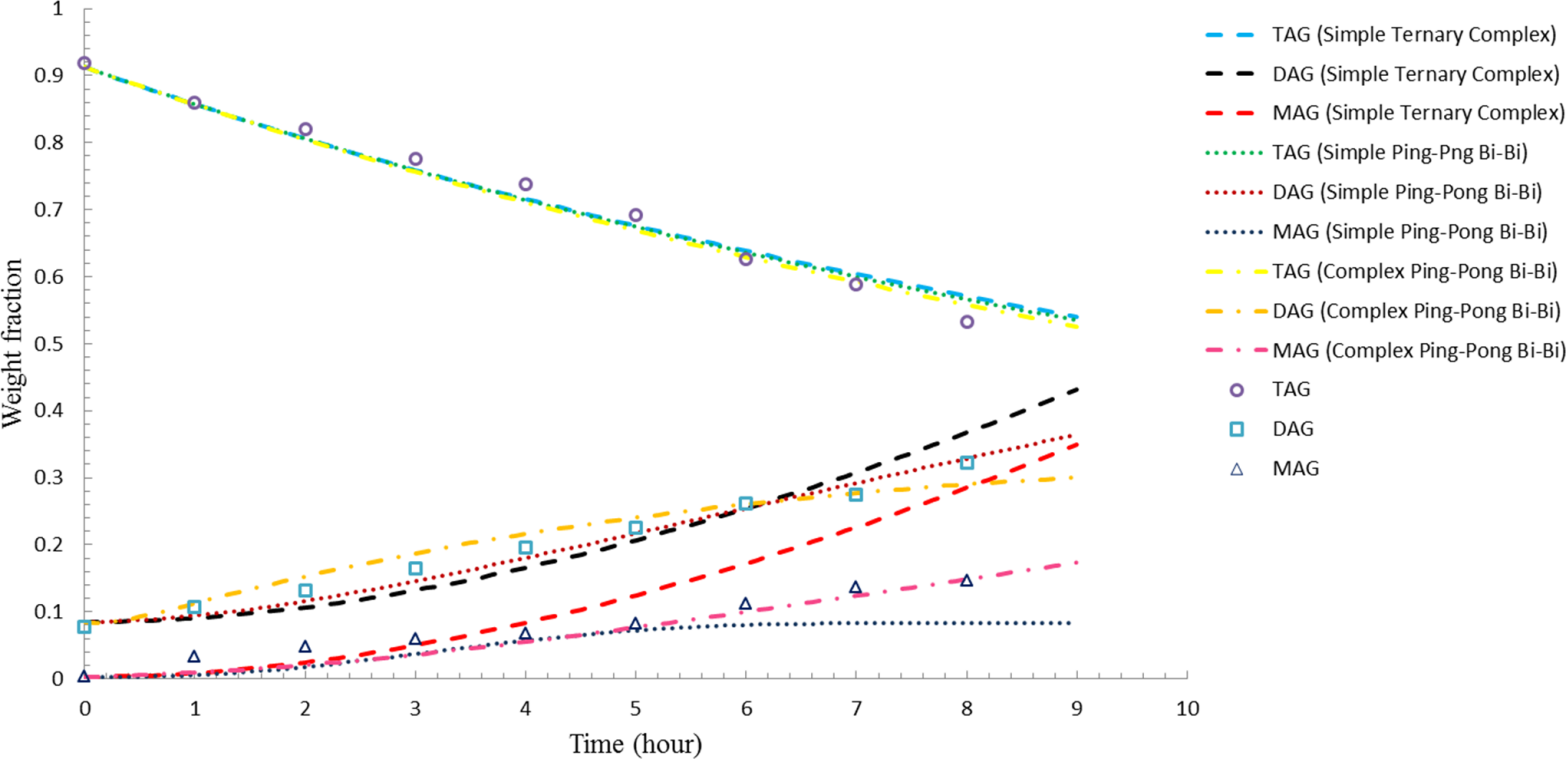
Comparison between simulated results of three different models and experimental data for enzyme load of 8 wt-% based on rate constant from Table 3.

The simulation results with the revised rate constants for 8 wt-% enzyme load for the three models were shown in Fig 4. The revised rate constants were now able to predict the results better for the 8 wt-% enzyme load. Again, the simulation results showed that the complex ping- pong bi-bi model performs better, looking at the fit of the kinetic model to the experimental results (Fig 4). Additional evidence that supports the validity of the complex ping-pong bi-bi model is the statistical analysis of the models (Table 3). Both SSE and RMSD were determined to be 0.0090 and 0.1638, respectively for complex ping-pong bi-bi mechanism. Chi-squared test was evaluated to be 3.8 x 10^−3^, indicating good agreement with observed experimental results. Overall, these simulation results suggested that the glycerolysis reaction followed the complex ping-pong bi-bi mechanism where the DAG was first produced prior to formation of MAG as the products. The DAG formed earlier then become a substrate for the formation of binary complex (E.DAG) and subsequently led to the formation of MAG.

Reaction constant or rate coefficient is crucial in chemical kinetics as it reveals the reaction velocity of each mechanism, giving an insight of a particular biochemical process. Based on the estimated rate constants for ping-pong bi-bi model, it can be concluded the formation of primary binary complex (E.TAG) is slow (low k_1_). It could be hypothesized that high energy barrier decreases the formation progress. Once the unstable primary complex was formed, it dissociates immediately into first product (DAG) and transforms into modified enzyme complex (E.FFA) instead of reforming the substrates (TAG and E) as k_3_ is several times higher than k_2_. Instantly, glycerol molecules show high affinity towards the modified enzyme complex and bind to it to become secondary binary complex (E.FFA.G) (high k_4_). This unsteady complex decomposes into stable MAG product without delay and enzyme particle is restored instead of following the reverse pathway for modified enzyme complex reformation (k_6_ >>> k_5_). The DAG formed earlier was then bound with enzyme to form enzyme complex (E.DAG) at slow rate due to low value of reaction coefficient k_7_. The unstable enzyme complex (E.DAG) would tend to reverse its reaction pathway in which both enzyme and DAG are regenerated instead of proceeding with the forward direction, producing more MAG and modified enzyme complex (E.FFA) (k_8_ > k_9_).

## Conclusions

The enzymatic glycerolysis reaction between TAG and G were successfully modeled based on simple ternary complex, simple ping-pong bi-bi and complex ping-pong bi-bi mechanisms. Based on the statistical analysis, complex ping-pong bi-bi model was proven to give a better and satisfactory agreement between experimental data and model results. Two sets of estimated rate constants were developed for the kinetic models, depending on the enzyme concentration. Accessibility of reactant mixtures to the active surface of the immobilized enzyme should be taken into consideration when enzyme loading is high (≥ 8 wt%) which was indicated by low k_1_ value. The present study provides valuable information and better understanding of the kinetic mechanism for lipase-catalyzed glycerolysis, allowing the use of the model for process optimization in near future.
